# Vaginal pH value can affect the susceptibility to human papillomavirus infection

**DOI:** 10.1186/s12879-024-09074-w

**Published:** 2024-02-09

**Authors:** Yinxia Liu, Zhengyu Li

**Affiliations:** 1 Department of Gynecology and Obstetrics, West China Second University Hospital, Sichuan University, No. 20 Section 3, Renmin South Road, Chengdu, Sichuan 610041 People’s Republic of China; 2 Department of Obstetrics and Gynecology, Key Laboratory of Birth Defects and Related Diseases of Women and Children, Ministry of Education, West China Second University Hospital, Sichuan University, Chengdu, People’s Republic of China

**Keywords:** HPV, HPV pseudovirus, pH value

## Abstract

**Background:**

Cervical cancer is the fourth most common cancer among women, with persistent high-risk human papillomavirus (HPV) infection being responsible for its progression. In healthy, pre-menopausal women, the vaginal pH value is maintained at 3.8–4.5, but various factors can affect it. Previous studies have suggested the relationship between vaginal pH value and HPV infection. In this study, we aimed to explore the relationship between vaginal pH and susceptibility of HPV infection.

**Methods:**

In our study, we retrospectively collected medical information from women who underwent leukorrhea examination at our hospital. We excluded women with infectious diseases or cancer, those who were pregnant or within 6 months post-delivery, and those without HPV test results within 6 months. The association between percentage of HPV infection and vaginal pH value was analyzed. Furthermore, we prepared HPV pseudovirus (PsVs) by co-transfecting structure plasmids and report plasmids in 293FT cells. In vitro, we changed the pH value of cell culture medium to investigate its influence on HPV PsVs infection. In vivo, we changed mouse’s vaginal pH value to investigate its influence on HPV PsVs infection.

**Results:**

Our retrospective study included 3115 women aged 20–78, including 2531 women with HPV negative and 584 women with HPV positive. The percentages of both HPV infection and high-risk HPV infection were higher in women with a vaginal pH value ≥5.0 compared to those with a pH value < 5.0. In vitro, HPV PsVs infection rate was higher in cell culture medium of higher pH value, dominantly due to the influence of pH value on the stage of HPV PsVs adhering to cell surface. Neither of the cell surface HPV receptors Syndecan-1 nor integrin α6 was found to be changed obviously in different pH values. In vivo, more HPV PsVs were adhered to the mouse’s vaginal epithelial cells with the increase of the vaginal pH value.

**Conclusions:**

Our study suggests a possible association between vaginal pH value and HPV infection. The pH value can influence the susceptibility of HPV PsVs infection by affecting the adhering of HPV PsVs to cells in vivo and in vitro. Additionally, the cell surface HPV receptors Syndecan-1 and Integrin α6 do not seem to be affected by pH value, and the specific mechanism needs to be further explored.

**Supplementary Information:**

The online version contains supplementary material available at 10.1186/s12879-024-09074-w.

## Introduction

According to Global Cancer Statistics 2020, cervical cancer ranked the fourth most common cancer among women, with 604,127 newly diagnosed cases and 341,831 deaths in 2020 [1]. In China, cervical cancer is the fifth most common cancer among women. In 2016, incidence rate of cervical cancer was 11.34/100,000 and mortality rate was 3.36/100,000 [2]. Persistent high-risk human papillomavirus (HPV) infection has been proved to be responsible for the progression of cervical intraepithelial neoplasia and cervical cancer [3, 4]. Among various high-risk HPV types, HPV 16 and HPV 18 are the most common high-risk HPV types found in cervical cancer [5, 6]. Risk factors for HPV infection includes aging, smoking, contraceptive using, bacterial vaginosis, immunodeficiency [7].

In healthy, pre-menopausal women, vaginal pH value maintains 3.8–4.5, and it is mainly determined by lactic acid produced from anaerobic glycolysis by Lactobacillus species [8]. Vaginal pH varied under various conditions, like menopause, menstruation, pregnancy, infection, medicine, etc. [9–11].

In 2012, Clarke et al. reported a prospective study concerning 9165 women aged 18–97. In up to 7 years’ follow ups, they found that detection of HPV infection was positively associated with vaginal pH [12]. Till now, only a few studies clarified the relationship between vaginal pH and HPV infection. In this study, we performed a retrospective study to analyze the relationship between vaginal pH value and HPV/high-risk HPV infection. We also conducted in vitro and in vivo experiments to explore the influence of pH value on HPV pseudovirus (PsVs) infection to cells and mouse’s vaginal epithelial cells.

## Methods

### Study population

The study was conducted in accordance with the Declaration Helsinki and approved by the Ethics Committee of West China Second University Hospital, Sichuan University. Women who underwent leukorrhea examination at West China Second University Hospital between February 1st, 2022 and April 30th, 2022, were selected, and their medical history and basic information were collected from the electronic medical records system and telephone follow-ups.

Using drying chemoenzymatic method, vaginal pH, one of the essential elements of leukorrhea examination, was recorded and stratified into two categories: < 5.0 and ≥ 5.0. HPV testing conducted within the last 6 months was also documented from the electronic medical records system and telephone follow-ups. Women who had infectious diseases or cancers, who were in pregnant or within 6 months after delivery, who had no HPV test results within 6 months were excluded from the study.

### Statistical analysis

Women were stratified into five categories based on their age: < 25 years, 25–34 years, 35–44 years, 45–54 years, and ≥ 55 years. Relationship between the percentage of HPV infection and pH value was analyzed in all women as well as in each age group. The odds of HPV positive (any type HPV infection, including low-risk HPV and/or high-risk HPV infection) were calculated. After eliminating women with pure low-risk HPV infection, who had only low-risk HPV infection, the odds of high-risk HPV positive (had at least one type high-risk HPV infection) were also calculated.

SPSS software version 20.0 (IBM Corp., Armonk, NY, USA) was used for statistical analysis. The Pearson chi-square test was used to compare categorical variables, and a *p*-value of < 0.05 was considered statistically significant.

#### Cells culture

Human cervical-cancer-derived cell lines Hela and Siha, human keratinocyte cell line HacaT, and virus packaging cell line 293FT, were acquired from American Type Culture Collection (Rockville, MD, USA) and were cultured in Dulbecco’s modified Eagle’s medium (DMEM) containing 10% fetal bovine serum (FBS) and 1% penicillin/streptomycin in a cell culture incubator with 5% CO2 at 37 °C.

#### HPV pseudovirus (PsVs) production

HPV 16 PsVs were produced in 293FT cells through the co-transfection of p16L1L2 and CLucf plasmids. The16L1L2 plasmid expresses the major protein L1 and L2, while the pCLucf plasmid expresses enhanced green fluorescence protein (EGFP). The plasmids pCLucf and p16L1L2 were obtained from Addgene (Cambridge, MA, USA). More detailed information on the production of HPV PsVs can be found in previous studies [13].

#### HPV PsVs infection

Hela, Siha, Hacat, and 293FT cells were cultured in 6-well plates overnight at a density of 2 × 10^5^ per well, then HPV16 PsVs was added and cultured at 37 °C for 72 h, so that plasmids p16L1L2 and pCLucf can enter into cells and express HPV 16 L1 protein and EGFP.

#### Influence of pH value on HPV16 infection under different treatment conditions

To investigate the effect of different pH values on HPV PsVs infection over time, viral adhering experiment and plasmid expression experiment were performed. In the viral adhering experiment, 293FT cells were incubated with HPV16 PsVs in DMEM with different pH values (6,7, and 8) for 2 h at 4 °C, followed by two washes with DMEM to remove unadhered PsVs. The cells were further incubated for another 70 h at 37 °C in DMEM with a pH value of 7.40, followed with flow cytometry analysis. Hela, Siha, and Hacat cells were lysed for western blot experiment after incubating for 2 h at 4 °C. In the plasmid expression experiment, 293FT, Hela, Siha, and Hacat cells were firstly incubated in DMEM with a pH value of 7.40 for 6 h at 37 °C, allowing encapsulated plasmids to enter the cells. The culture medium was then changed to DMEM with different pH values (6,7, and 8), and the cells were incubated for another 64 h at 37 °C. For Hela, Siha, and Hacat cells, cell lysis was performed for western blot analysis. For 293FT cells, flow cytometry was used for analysis.

#### HPV PsVs infection detection

In 293FT cells, the expression of EGFP in 293FT cells was detected using flow cytometry. In the viral adhering experiment, PsVs adhered on cells were compared through western blot assay by monitoring HPV 16 L1 protein in Hela, Siha, and Hacat cells after 2 h infection. In the plasmid expression experiment, the amount of virus that had entered into Hela, Siha, and HacaT cells was monitored through the detection of HPV16 L1 protein using a western blot assay after a 72-hour infection.

#### Flow cytometry

Flow cytometry was performed to analyze both total and HPV PsVs-infected 293FT cells. Prior to viral infection with HPV16 PsVs, 293FT cells were cultured in 6-well plates overnight at a density of 2 × 10^5^ cells per well. After 72 hours of viral infection, flow cytometry was employed to detect the number of cells infected with HPV16 PsVs, as indicated by the expression of EGFP. In brief, the cells were washed with PBS, trypsinized, and then centrifuged at 500 g for 3 minutes at 4 °C. Subsequently, the cells were resuspended in PBS for flow cytometry analysis. The analysis was conducted using a FACS Calibur instrument (Millipore, Germany). For statistical analysis, the means ± standard deviations (SD) were calculated based on the statistics from three independent experiments. The Student’s t-test was utilized for the statistical analysis.

#### Western blot

Cells were washed with PBS and total protein was harvested using RIPA lysis buffer (Beyotime, China). 20ul denatured protein was separated by sodium dodecyl sulfate-polyacrylamide gel electrophoresis (SDS- PAGE, GenScript, USA) and transferred onto polyvinylidene fluoride membranes (PVDF, Millipore, USA). After blocking with 5% non-fat milk for 1 h at room temperature, the membranes were incubated with primary antibodies (HPV16-L1-specific antibody CamVir1, Abcam, Cambridge, MA, USA, 1:3000 and anti-β-actin, Bioss, China, bs-0061R, 1:1000) at 4 °C overnight. The membranes were washed and exposed to corresponding horseradish peroxidase (HRP)-conjugated goat anti-rat (1:5000, Mengbio, China, MS002A) or goat anti-rabbit (1:1000, Beyotime, China, A0208) secondary antibodies diluted in TBST buffer for 2 h at room temperature. Finally, the protein bands were visualized with an enhanced chemiluminescence (ECL) kit (Millipore, USA).

#### Animal study

Fifteen 6-week-old BALB/c mice were divided into three groups (five in each group). 4 days before genital challenge, 3mg medroxyprogesterone was injected subcutaneously each mouse. On the fifth day, after anesthesia with 50 mg/kg pentobarbital calcium, the mouse’s genital tract was brushed clockwise and counterclockwise using a cytobrush to physically damage the mouse’s vaginal epithelium. Then 50ul 4% nonoxynol-9 (N-9) was poured into the vagina to chemically damage the mouse’s vaginal epithelium. HPV 16 PsVs was prepared by mixing with 4% carboxymethylcellulose (CMC) in 3:1. Six hours later, PBS with different pH value (pH = 6, pH = 7, and pH = 8) was used to wash the mouse’s vagina to form different pH conditions. After three times PBS washing, 20ul HPV 16 PsVs and 4% CMC mixture was poured into each mouse’s vagina. 2 hours later, the mice vaginal tissues were dissected and frozen sections were made. Indirect fluorescence immunoassay was used to label HPV PsVs adhered to mouse vaginal epithelium to observe the influence of pH value on HPV PsVs adhering to mice vaginal epithelium cells.

### Histological analysis

The reproductive tract tissue samples were frozen in OCT freezing media (Electron Microscopy Sciences) and cut into 5–7-mm-thick sections. The slides were fixed in a 2% paraformaldehyde/PBS solution for 10 min, followed by incubation with a rabbit polyclonal serum against HPV16L1 at 1:1000 in PBS for 1 h at room temperature. Then the slides were incubated with a Cy3- conjugated anti-rabbit secondary antibody for 1 h at room temperature away from light. The slides were then washed in PBS and mounted with a DAPI containing antifade mounting solution (Prolong Gold; Thermo Fisher Scientific). Imaging was performed using a Nikon confocal microscope.

## Results

### Clinical retrospective study

**Fig. 1:**
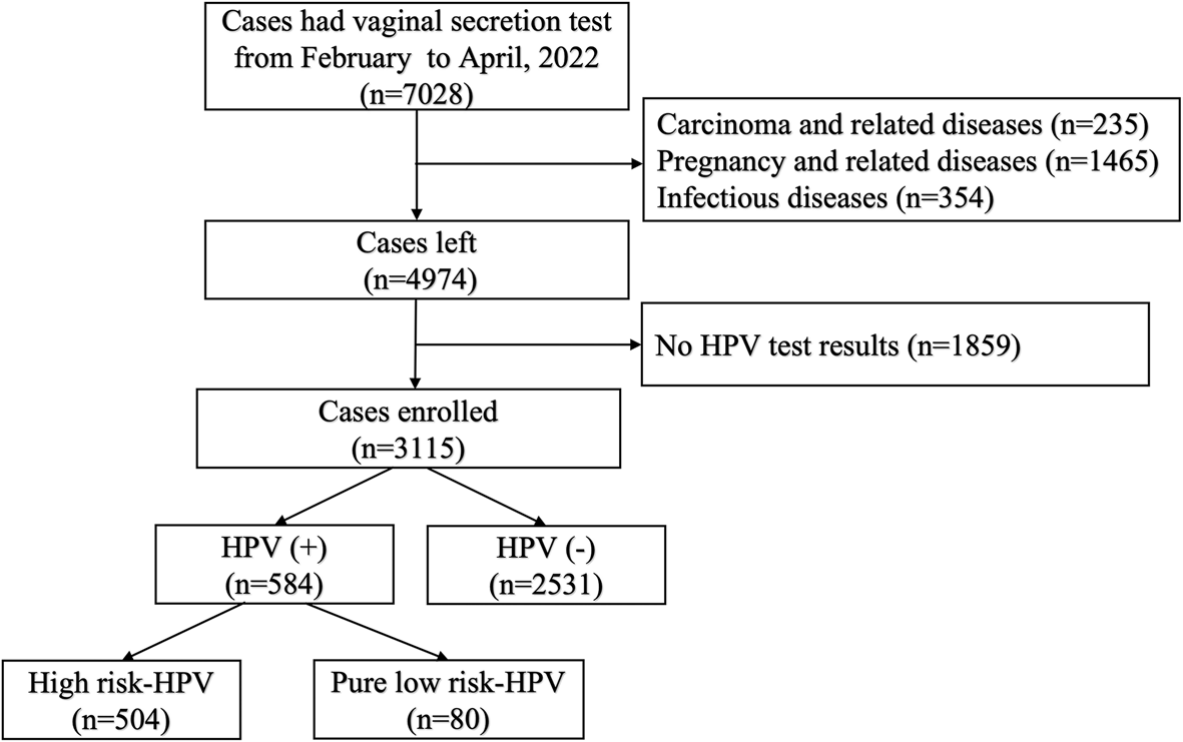
Flow chart illustrating the patient selection procedure

From February 1st to April 30th, 2022, a total of 7028 women received leukorrhea examination at West China Second University Hospital, and 3115 women were included in this study, including 2531 women of HPV negative and 584 women of HPV positive. 80 cases were pure low-risk HPV positive and at least one type of high-risk HPV infection was detected in the rest 504 individuals (Fig. 1).

For all women enrolled, percentage of HPV infection was higher in patients with a vaginal pH value≥5.0 than those with a vaginal pH value < 5.0. In each age group, the percentage of positive HPV cases was also higher in women with a vaginal pH value ≥5.0 than those with a vaginal pH value < 5.0, particularly in women aged 25–34 and 35–44 (Table 1).

**Table 1:** Relationship between vaginal pH value and positive HPV

Vaginal pH	Positive HPV	OR (95% CI)	*P*-value	Positive high-risk-HPV	OR (95% CI)	*P*-value
all women						
< 5.0	17.92%	Ref	< 0.001	15.82%	Ref	< 0.001
≥ 5.0	27.51%	1.535 (1.245–1.893)		25.00%	1.774(1.316–2.391)	
≤25 years						
< 5.0	22.12%	Ref	0.175	18.52%	Ref	0.523
≥ 5.0	45.45%	2.933 (0.826–10.416)		33.33%	2.200(0.507–9.554)	
25–34 years						
< 5.0	16.13%	Ref	0.047	14.17%	Ref	0.028
≥ 5.0	23.58%	1.605 (1.002–2.569)		22.12%	1.720(1.056–2.800)	
35–44 years						
< 5.0	16.09%	Ref	< 0.001	14.98%	Ref	0.002
≥ 5.0	33.90%	2.674 (1.513–4.727)		30.36%	2.475(1.357–4.513)	
45–54 years						
< 5.0	21.45%	Ref	0.442	17.62%	Ref	0.195
≥ 5.0	25.81%	1.274 (0.686–2.365)		24.59%	1.525(0.803–2.896)	
≥55 years						
< 5.0	33.80%	Ref	0.619	29.85%	Ref	0.458
≥ 5.0	37.88%	1.194 (0.593–2.403)		35.94%	1.318(0.635–2.739)	

After eliminating 80 cases of pure low-risk HPV infection, an analysis of the remaining 504 high-risk HPV positive cases demonstrated the percentage of high-risk HPV infection was also positively associated with vaginal pH value. In each age group, the percentage of positive high-risk HPV cases was higher in women with a vaginal pH value ≥5.0 than those with a vaginal pH value < 5.0, particularly in women aged 25–34 and 35–44 (Table 1).

### Effects of pH value on HPV PsVs infection in vitro

**Fig. 2:**
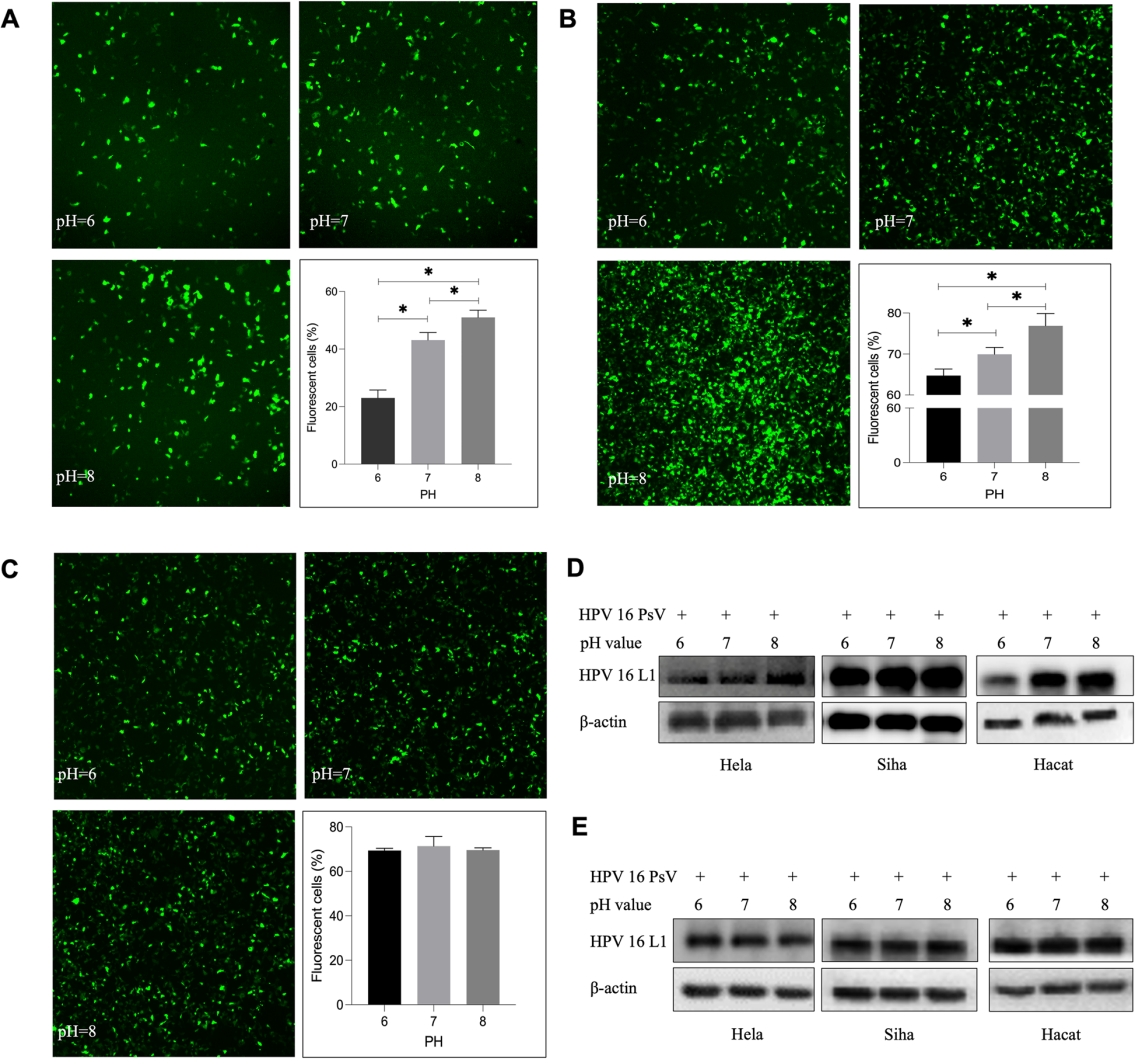
Influence of pH value on HPV16 infection in cells. **A** Influence of pH value on HPV16 infection in 293FT cells. **B** Influence of pH value on HPV16 infection in 293FT cells in viral adhering experiment. **C** Influence of pH value on HPV16 infection in 293FT cells in plasmid expression experiment. **D** Influence of pH value on HPV16 infection in Hela, Siha, and Hacat cells in viral adhering experiment. **E** Influence of pH value on HPV16 infection in Hela, Siha, and Hacat cells in plasmid expression experiment

DMEM medium was adjusted to pH values of 6, 7, and 8 by titrating sterile lactic acid and sodium hydroxide into DMEM of pH = 7.40. After incubating 293FT cells in DMEM of different pH values for 72 hours at 37 °C, the cells were trypsinized for flow cytometry. The results demonstrated that the percentage of infected 293FT cells increased with elevated pH value (pH = 6, pH = 7, pH = 8) (Fig. 2A).

### Influence of pH value on HPV16 infection under different treatment conditions

**Fig. 3:**
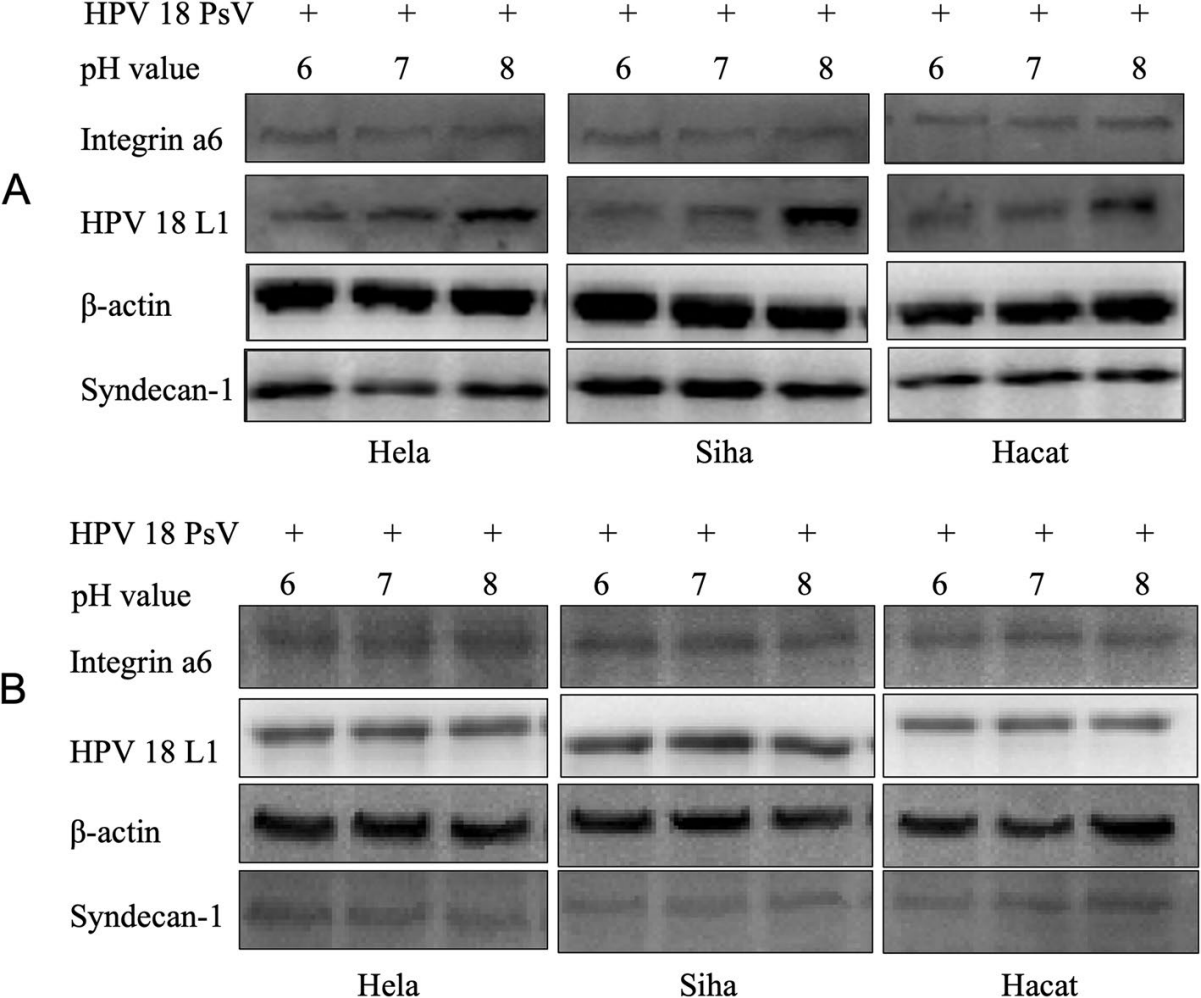
Influence of pH value on HPV18 infection in cells. **A** Influence of pH value on HPV18 infection in cells in viral adhering experiment. **B** Influence of pH value on HPV18 infection in cells in plasmid expression experiment

To determine the time period during which pH value affects HPV PsVs infection, viral adhering and plasmid expression experiments were performed. In the viral adhering experiment, 293FT cells were incubated with HPV16 PsVs in DMEM of different pH values for 2 hours at 4 °C, allowing the HPV 16 PsVs adhering to cell surfaces instead of entering them. The culture medium was then changed to DMEM of pH 7.40. 72 h after infection, flow cytometry results indicated that the number of adhered HPV PsVs increased in cultures with higher pH values (Fig. 2B). For Hela, Siha, and Hacat cells, after HPV PsVs infection, cells were lysed for western blotting analysis following a 2-hour incubation at 4 °C to compare the number of adhered PsVs. The results showed that more HPV 16 PsVs were adhered to the cells when incubated in culture medium with higher pH values (Fig. 2D). In the plasmid expression experiment, the culture medium was changed to the medium of different pH values after incubating the cells and HPV 16 PsVs in DMEM of pH 7.40 for 6 hours at 37 °C to study the influence of pH value on protein expression after encapsulated plasmids enter cells. The results revealed significant difference in EGFP expression in 293FT cells (Fig. 2C) and HPV 16 L1 protein expression in Hela, Siha, and Hacat cells (Fig. 2E) under different pH values. HPV 18 PsVs were prepared using the same method, and the results demonstrated a similar trend as HPV 16 PsVs (Fig. 3A and B).

To primarily explore the mechanism by which pH value affects HPV PsVs infection, cell surface HPV receptors syndecan-1 and Integrin α6 were analyzed. In the viral binding experiment, different pH values resulted in different levels of HPV infection, while no significant differences were observed in cell surface HPV receptors syndecan-1 and integrin α6 (Fig. 3A). In the plasmid expression experiment, different pH values had no discernible effect on either protein expression inside cells or the expression of HPV receptors on the cell surface (Fig. 3B).

### Effects of pH value on HPV PsVs infection in vivo

**Fig. 4:**
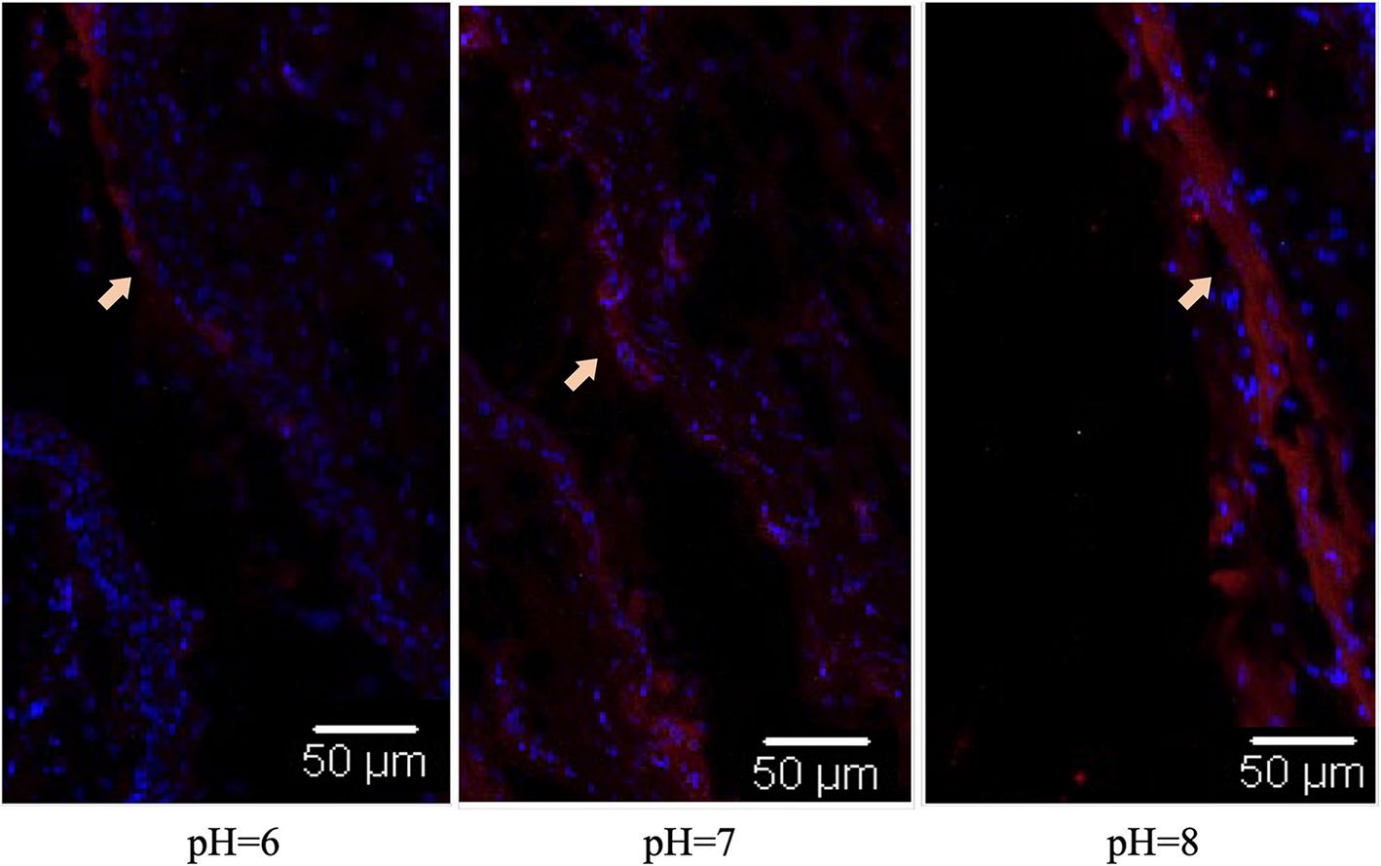
Influence of pH value on HPV PsVs adhering on mice vaginal epithelial cells

The vaginal pH value of BALB/c mice was neutral or alkaline. To investigate the influence of pH value on HPV 16 PsVs infection in vaginal epithelial cells of BALB/c mice, three washes with PBS of different pH values (pH = 6, pH = 7, and pH = 8) were performed in different groups before HPV 16 PsVs infection. The results showed that more HPV 16 PsVs adhered to the vaginal epithelial cells of mice when PBS with a higher pH value was used before infection (Fig. 4).

## Discussion

Persistent infection of high-risk HPV is a major risk factor for the development of cervical cancer [3, 4]. Previous studies have explored several high-risk factors for HPV infection, including frequent sexual activity, multiple sexual partners, smoking, oral contraceptive use, etc. [7]. In recent years, increasing studies have identified there is also a correlation between HPV infection and vaginal microbiota [14–16].

Normally, the vaginal pH is maintained between 3.8 to 4.5, primarily through the action of lactobacilli, which convert glycogen in the vaginal squamous epithelial cells into lactic acid, maintaining a slightly acidic environment in the vagina to resist microbial invasion [8]. However, several factors can cause changes in vaginal pH, including the menstrual cycle, menopause, vaginal douching, prolonged use of antibiotics, frequent sexual activity, and the use of contraceptive condoms with alkaline lubricants [9–11]. Bacterial vaginitis (BV) induces a decrease in the abundance of lactobacilli and an increase in the abundance of facultative anaerobes and anaerobes, which also leads to an increase in vaginal pH value [17].

In a prospective study published in 2012 on the relationship between vaginal pH and HPV infection, over 9000 women were enrolled and followed up for a total of over 20,000 times. The study recorded the vaginal pH of all included women and followed them for up to 7 years. The final results revealed that during the follow-up period, the rate of HPV infection was significantly higher in women with higher vaginal pH compared to those with lower vaginal pH at enrollment. This large-scale prospective study provided evidence for the relationship between higher vaginal pH and a higher rate of HPV infection [12]. Our retrospective clinical study also found that the vaginal pH value in women increases with age, and there is a correlation between vaginal pH value and HPV infection, as well as high-risk HPV infection.

In studies on HIV, it has been proven that the acidity of cervical vaginal secretions usually provides protection against HIV [18]. In an in vitro experiment, activity of HIV particles was completely and irreversibly inhibited when incubated at pH < 5.4 for 20 minutes or pH < 5.7 for 2 hours at 37 °C [19]. Additionally, semen retained in the vagina after sexual intercourse can increase vaginal pH, facilitating the transmission of HIV between men and women [20]. Our in vivo and in vitro experiments confirmed that the infection rate of HPV PsVs to both cells and mouse vaginal epithelial cells increases with the elevation of pH values, and pH value primarily affects the adhering of HPV PsVs to cell surfaces.

Heparan sulfate proteoglycans (HSPGs) and integrins are widely studied receptors for HPV infection. HSPGs on membrane include two major families: syndecans, which are proteoglycans, and glypicans, which are glycosylphosphatidylinositol-anchored proteins. Studies have shown that HSPGs are involved in the infection process of various viruses, including HPV, HIV, herpes simplex virus, dengue virus, and foot-and-mouth disease virus [21]. In other studies, Integrin α6 has been found to be the major receptor for HPV 16 infection [22]. Our research has found that different pH values primarily affect the adhering of HPV pseudovirus to the cell surface, while changes of pH value does not affect the expression of HPV receptors syndecan-1 and integrin α6 on the cell surface. Further research is needed to explore the mechanisms that pH value affects the HPV PsVs infection.

According to the Global Cancer Statistics 2020, cervical cancer is the fourth most common malignancy among women [1] and persistent infection of high-risk HPV is a major risk factor for the development of cervical cancer. In the study, we discovered that there is a possible association between vaginal pH value and HPV infection. As vaginal pH value can be affected by a number of factors, abandoning habits and curing diseases those may elevating vaginal pH value may play an essential role in preventing HPV infection. Our study also found that pH value can influence susceptibility of HPV PsVs infection by affecting the adhering of HPV PsVs to cells. Along with the study of HIV [19], it may due to the direct effect of pH value on HPV PsVs. Our team are working on it to further study the mechanism.

### Supplementary Information

**Additional file 1.**

## Data Availability

The datasets generated or analyzed during this study are available from the corresponding author on reasonable request.
