# Supplementary figures and images for: Vaginal pH value can affect the susceptibility to human papillomavirus infection
Source: BMC Infect Dis. 2024 Feb 9;24:176. doi: 10.1186/s12879-024-09074-w (PMC10854022; doi:10.1186/s12879-024-09074-w)

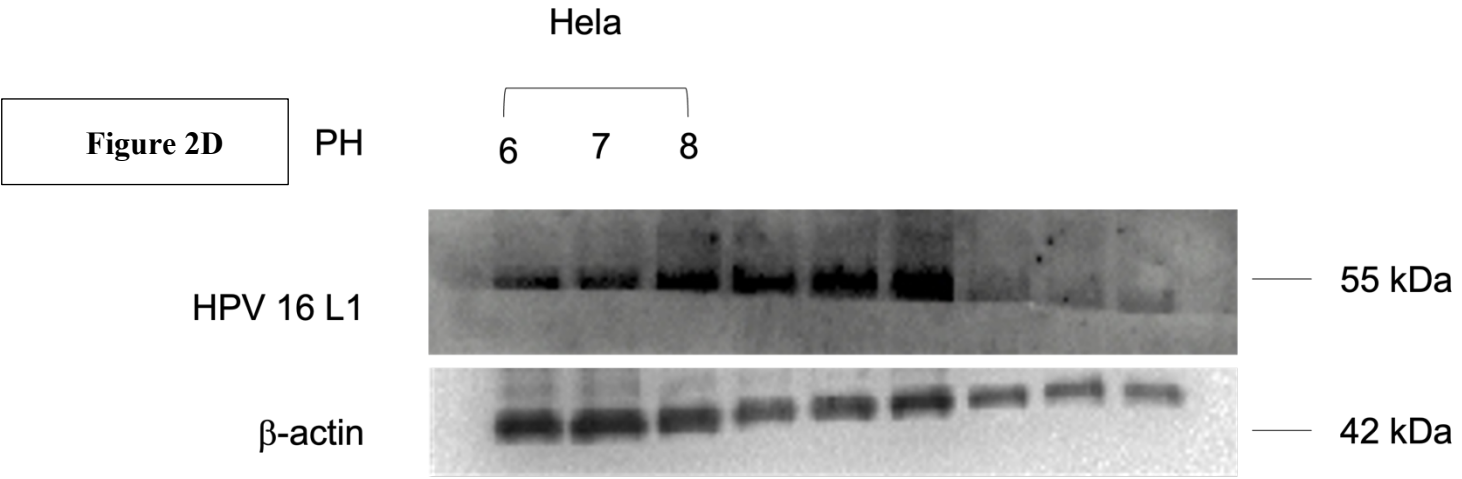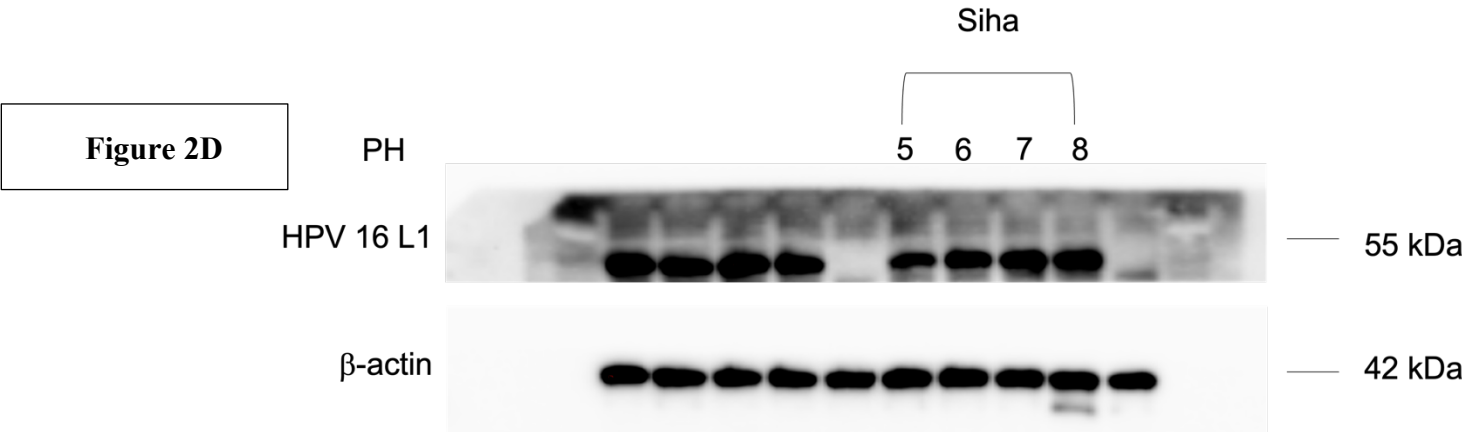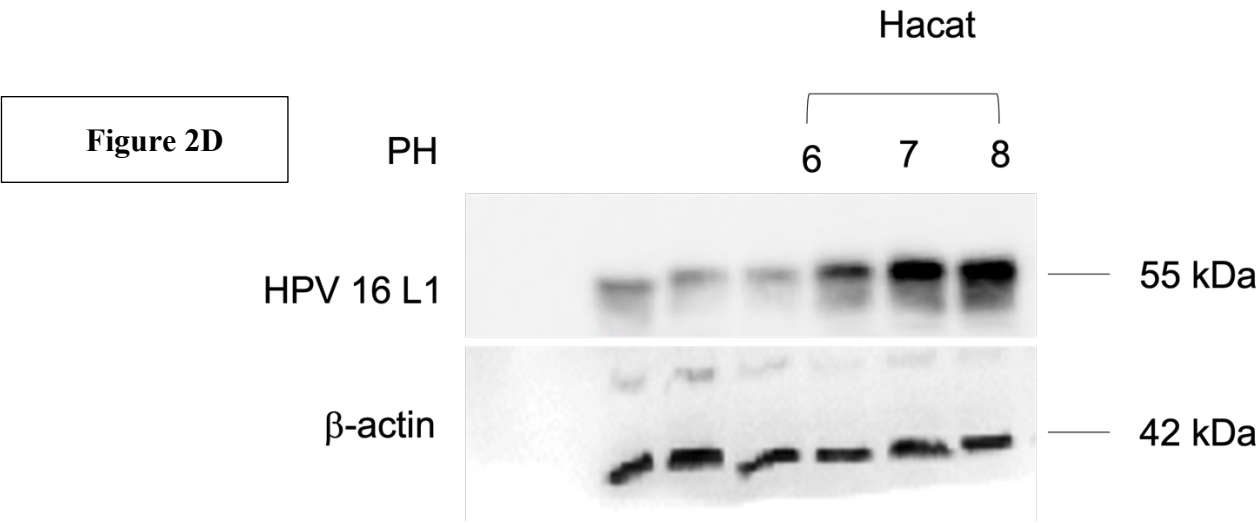

Figure 2E

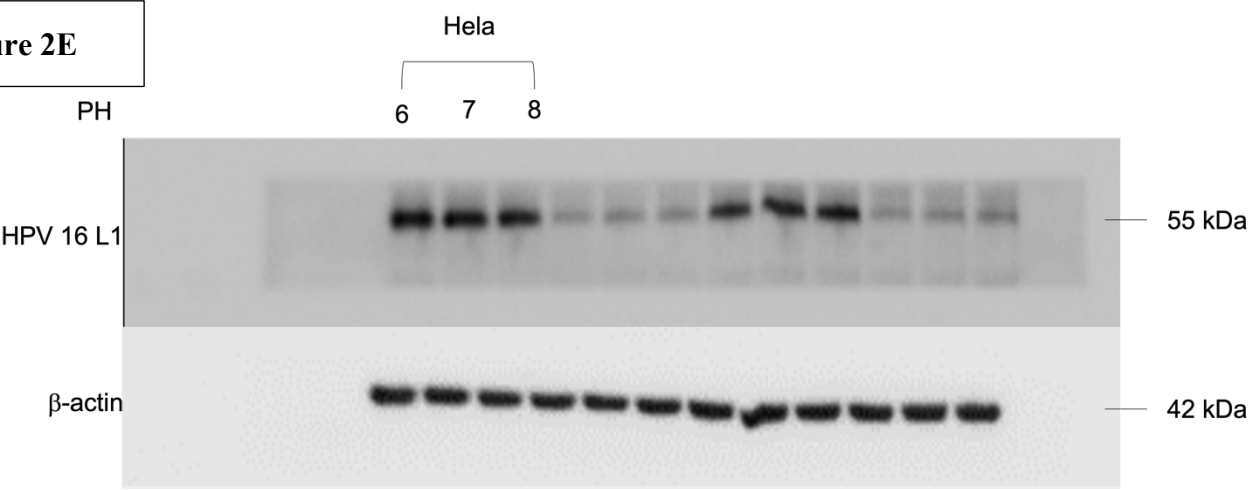

Figure 2E

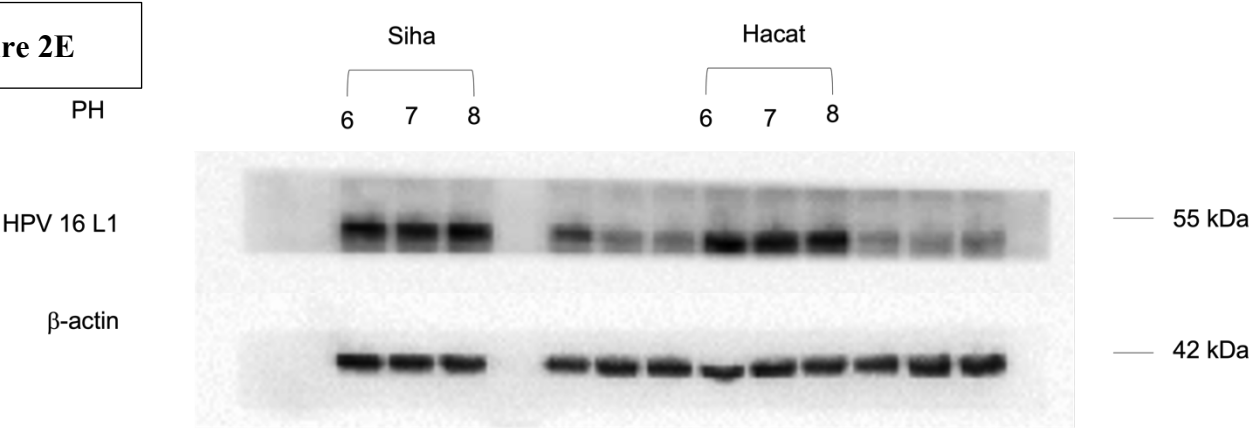

**Figure 3A**

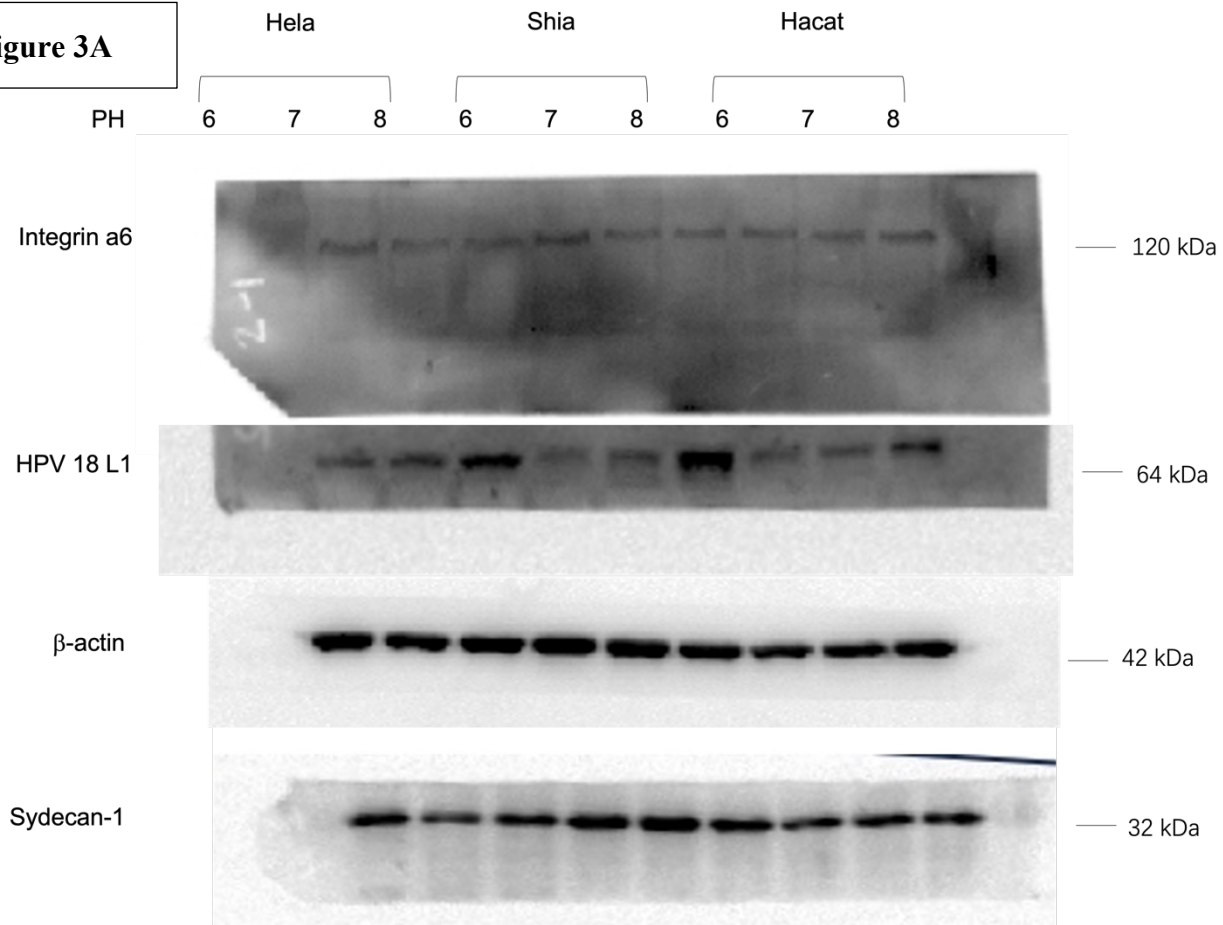

**Figure 3B**

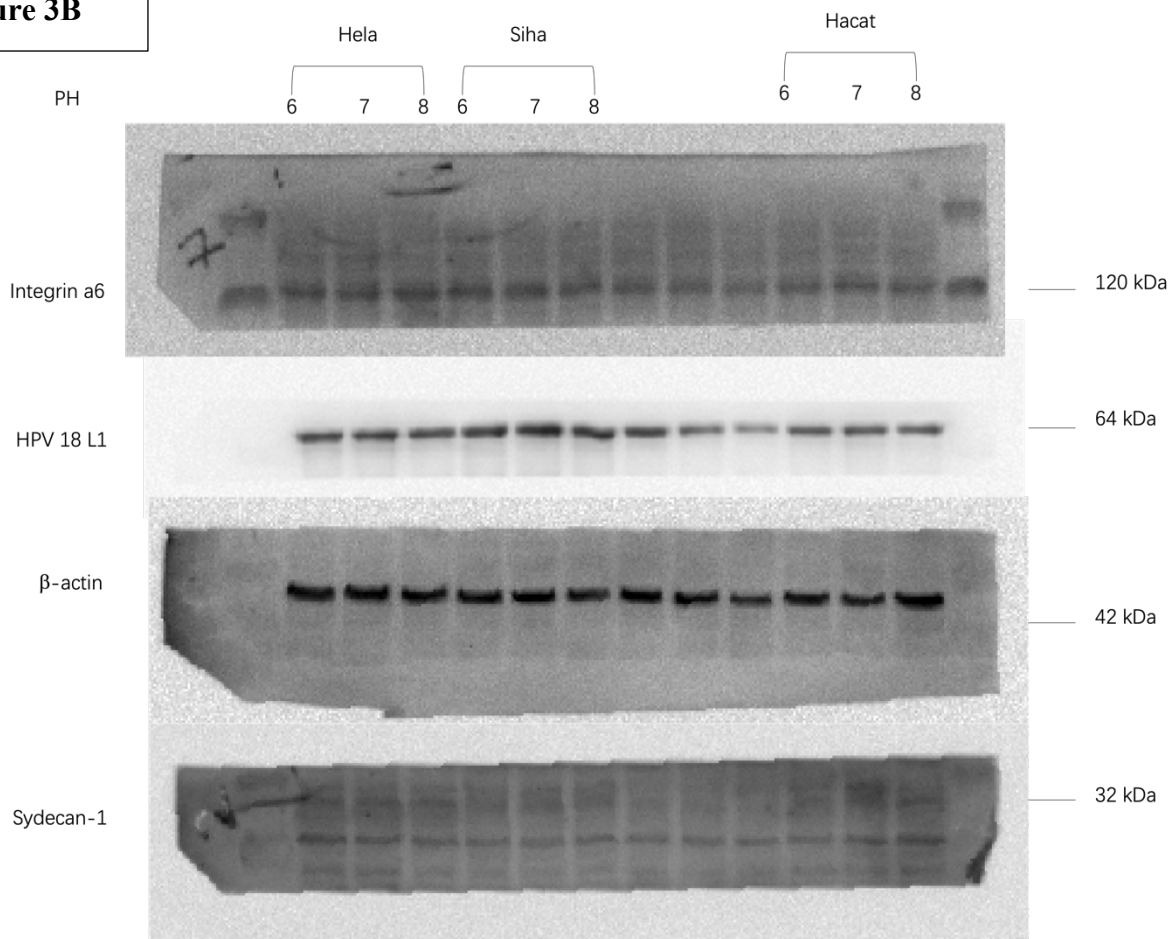

Supplement: Supplementary file 1 — Additional file 1. [file 12879_2024_9074_MOESM1_ESM.pdf]
